# Immunophenotypic Stratification of Primary Sjögren’s Syndrome Reveals Distinct Lymphocyte Profiles and Clinical Manifestations

**DOI:** 10.1155/jimr/9295560

**Published:** 2026-02-26

**Authors:** Yimeng Jia, Sicheng Huang, Ye Guo, Anqi Wang, Chuiwen Deng, Yunyun Fei

**Affiliations:** ^1^ Department of Rheumatology and Clinical Immunology, Peking Union Medical College Hospital, Peking Union Medical College, Chinese Academy of Medical Sciences, #1 Shuai-Fu-Yuan, Dongcheng District, Beijing, 100730, China, cacms.ac.cn; ^2^ The Ministry of Education Key Laboratory, National Clinical Research Center for Dermatologic and Immunologic Diseases (NCRC-DID), Beijing, China; ^3^ Department of Clinical Laboratory, State Key Laboratory of Complex Severe and Rare Diseases, Peking Union Medical College Hospital, Chinese Academy of Medical Science and Peking Union Medical College, Beijing, China, cams.ac.cn; ^4^ Department of Heath Medicine, Peking Union Medical College Hospital, Peking Union Medical College, Chinese Academy of Medical Sciences, Beijing, China, cacms.ac.cn

**Keywords:** clinical stratification, lymphocyte subset, primary Sjögren’s syndrome

## Abstract

**Objective:**

Primary Sjögren’s syndrome (pSS) is a heterogeneous autoimmune disorder with diverse clinical manifestations and limited effective therapies. This study aimed to stratify pSS patients into distinct immunophenotypic subgroups based on peripheral lymphocyte profiles and to explore the clinical relevance of these subgroups, thereby informing personalized management for pSS.

**Methods:**

A retrospective cohort of 133 Chinese pSS patients and 241 age‐ and sex‐matched healthy controls (HCs) was analyzed. Immunophenotyping of 11 lymphocyte subsets was performed using flow cytometry in peripheral blood. K‐means clustering was employed to identify patient subgroups based on lymphocyte profile. Clinical data were collected and compared across clusters.

**Results:**

Lymphocyte profiles in pSS patients differed from those in HCs, featuring elevated proportions and estimated counts of activated CD8^+^ T cells along with reduced naïve CD4^+^ T cells and NK cells. Unsupervised clustering analysis identified three distinct patient subgroups based on immunophenotypic patterns: Cluster 1 was characterized by significantly higher proportions of CD8^+^ T cells and showed more frequent hematologic and serological abnormalities, including higher rates of hyperglobulinemia, anti‐Ro52 positivity, and high‐titer ANA positivity. Cluster 2 was distinguished by higher NK and B cell proportions and clinically presented with greater pulmonary and hepatic involvement along with higher disease damage scores. Cluster 3 maintained lymphocyte distributions closest to HCs but exhibited more frequent constitutional symptoms and cutaneous involvement coupled with lower serological activity.

**Conclusion:**

Lymphocyte profiling may help stratify pSS patients into clinically distinct subgroups, potentially corresponding to different pathobiological endotypes, and could thus inform patient stratification.

## 1. Introduction

Primary Sjögren’s syndrome (pSS) is a chronic autoimmune disorder characterized by a broad clinical spectrum, ranging from exocrine gland dysfunction to systemic manifestations affecting the joints, lungs, kidneys, liver, nervous, and musculoskeletal systems [1]. Existing research has reported limited associations between patient‐reported symptoms and objective dryness measures or systemic involvement [2]. Moreover, targeted immunomodulatory therapies for pSS have largely failed in clinical trials [3–6], partly due to the disease’s marked heterogeneity [7, 8].

Given the heterogeneous nature of pSS, substantial efforts in recent years have focused on identifying distinct pSS subgroups, highlighting the critical need for precise stratification and personalized therapies [9–11]. Previous studies have proposed classifications based on serological markers [12], histopathological features of salivary glands [13], interferon (IFN) signaling [14], or patterns of systemic involvement [15]. Moreover, unsupervised clustering algorithms have emerged as a powerful tool for uncovering clinically relevant subgroups by analyzing unlabeled multidimensional data [16, 17]. Transcriptomic and clinical clustering studies further support the utility of this approach in dissecting pSS heterogeneity [11, 18].

Lymphocyte subset analysis is a widely used clinical tool for assessing immune function [19]. In autoimmune diseases, it has proven valuable for delineating disease endotypes and predicting clinical outcomes [20]. In pSS, studies have identified patient clusters linked to activated CD4/CD8 T‐cell signatures, disease activity, and glandular inflammation, underscoring the role of immune phenotyping in clarifying disease heterogeneity [21]. Building on these insights, we hypothesized that immune cell profiling could facilitate the stratification of pSS patients, thereby helping to clarify the inherent heterogeneity of pSS and inform more precise therapeutic strategies. In this study, we applied cluster analysis to a cohort of Chinese pSS patients and identified three immunophenotypically distinct subgroups, each exhibiting unique clinical features. Our findings may inform the development of future therapeutic interventions tailored to specific immune‐pathological signatures in pSS.

## 2. Method

### 2.1. Study Participants

This retrospective study was conducted at Peking Union Medical College Hospital (PUMCH) between January 2022 and December 2024. Patients diagnosed with pSS and possessing at least one peripheral immune cell test result were eligible for inclusion. Exclusion criteria encompassed: (1) patients with secondary Sjögren’s syndrome associated with other connective tissue diseases; and (2) patients who had received prednisone ≥7.5 mg/day or equivalent therapy [22] or immunosuppressants, including cyclophosphamide (CYC), mycophenolate mofetil (MMF), tacrolimus (FK506), or cyclosporine A (CsA), et cetera. [23]. A final cohort of 133 patients with pSS, fulfilling the 2016 American College of Rheumatology (ACR)/European League Against Rheumatism (EULAR) classification criteria [24] or the 2012 ACR classification criteria [25], was included in the analysis. Concurrently, 241 healthy controls (HCs), matched for age and sex with the pSS patients, were recruited. The study protocol adhered to the principles outlined in the Declaration of Helsinki and received formal approval from the Institutional Ethics Committee (I‐24PJ1657).

### 2.2. Lymphocyte Count and Subset Analysis

Isolated PBMCs from fresh EDTA‐anticoagulated blood were pretreated with Fc receptor blocking solution (BioLegend, 1:20) for 10 min and subsequently stained with surface antibodies (BioLegend, 1:50) for 20 min at room temperature in the dark. Three staining panels were utilized: a panel for T, B, and NK cell subsets (CD3‐FITC, CD4‐APC‐Cy7, CD56‐APC, CD16‐APC, CD19‐PE, and CD8‐PerCP‐Cy5.5); a panel for CD4^+^ T cell subsets (CD3‐FITC, CD4‐APC, CD28‐PE‐Cy7, CD45RA‐PerCP‐Cy5.5, and CD62L‐PE); and a panel for CD8^+^ T cell subsets (CD3‐FITC, CD8‐PerCP‐Cy5.5, HLA‐DR‐APC, CD38‐APC‐Cy7, CD28‐PE‐Cy7). Matched isotype controls were included. Cell acquisition was performed using a FACSAria II flow cytometer (BD Biosciences), and data analysis was conducted with FlowJo software. The gating strategy is detailed in Supporting Information 1: Figure S1. Estimated counts were calculated by multiplying the lymphocyte count from routine blood analysis (SYSMEX XT‐1800i) of the same specimen by the subset frequency derived from flow cytometric analysis.

### 2.3. Clinical Data Collection

Demographic and clinical data at enrollment included patient age, sex, disease duration, clinical symptoms, laboratory indicators, EULAR Sjogren’s Syndrome Disease Activity Index (ESSDAI) [26], EULAR Sjogren’s Syndrome Patient Reported Index (ESSPRI) [27], Sjogren’s Syndrome Disease Damage Index (SSDDI; six domains were assessed including oral/salivary damage, ocular damage, neurologic damage, pleuropulmonary damage, renal impairment, and lymphoproliferative disease) [28]. Constitutional symptoms include fever or weight loss caused by pSS. Cutaneous manifestations in our cohort (e.g., erythema, purpura, and panniculitis) were confirmed by rheumatologists after excluding secondary causes such as infection or allergy. Pulmonary involvement was defined as interstitial lung disease on high‐resolution computed tomography (HRCT). Liver injury was diagnosed per established criteria: either (1) alanine aminotransferase (ALT) or conjugated bilirubin (CB) >2 × upper limit of normal (ULN), or (2) concurrent elevations of aspartate aminotransferase (AST), alkaline phosphatase (ALP), and total bilirubin (TB), with at least one parameter >2 × ULN [29]. A board‐certified rheumatologist confirmed all diagnoses after excluding non‐pSS causes of hepatic dysfunction.

### 2.4. Sparse Partial Least Squares Discriminant Analysis (sPLS‐DA)

sPLS‐DA was performed using the package in R [30]. A 10‐repeat, five‐fold cross‐validation prevented overfitting (Supporting Information 1: Figure S2). The optimal number of components was selected for model tuning. Sample separation was shown by projecting data onto the first two components. Prediction intervals were based on 95% confidence ellipses. The top 10 variables were identified and visualized using loading plots.

### 2.5. *K*‐Means Clustering

*K*‐means clustering was performed using the stats package in R to stratify patients with pSS (Supporting Information 2: Data Table). The analysis was based on 11 immune phenotypic variables, including the frequencies of CD3^+^ T cells, CD19^+^ B cells, CD3^−^(CD56^+^CD16)^+^NK cells, CD3^+^CD4^+^ T cells, CD3^+^CD8^+^ T cells, CD4^+^CD45RA^−^ T cells, CD4^+^CD45RA^+^CD62L^+^ T cells, CD4^+^CD28^+^ T cells, CD8^+^CD28^+^ T cells, CD8^+^HLA‐DR^+^ T cells, and CD8^+^CD38^+^ T cells. Demographic and clinical parameters were excluded from the clustering. The optimal number of clusters was determined by the elbow method after standardizing the data [31]. The algorithm was run with 50 random starts and a fixed random seed to ensure reproducibility.

### 2.6. Statistical Analysis

Data were analyzed using SPSS version 19 (SPSS Inc., Chicago, IL, USA). The normality of the data was assessed using the Shapiro–Wilk test. Quantitative data were presented as mean ± standard deviation (SD) for normally distributed data or median with interquartile range (IQR) for non‐normally distributed data. For categorical variables, we employed the chi‐square test or Fisher’s exact test. For comparisons between two groups, Mann–Whitney *U*‐tests were used. For comparisons involving more than two groups, the Kruskal–Wallis test followed by Dunn’s post hoc test was applied. Univariate logistic regression analysis was performed and displayed by forest plots.

## 3. Result

### 3.1. Clinical Profile of pSS Patients

Table 1 and Supporting Information 1: Table S1 depicted the clinical profiles of pSS patients in our study. The cohort comprised 133 patients with pSS, the majority of whom were female (96%), with a mean age of 48.3 ± 15.0 years and a median disease duration of 4 (1–8) years. Seventy‐seven percent experienced dry mouth and 59% had dry eyes. Fatigue and constitutional symptoms (fever and weight loss) were present in 20% and 14% of patients, respectively. The average ESSDAI score among patients was 2 (0–5), indicating that those with mild disease activity constituted the majority. The most common extra‐glandular manifestations in our cohort were hematological (23%), cutaneous (20%), and articular (14%), followed by pulmonary (11%) and renal (9%) involvement. Additionally, 13% of patients had liver injury.

**Table 1:** Clinical characteristics of pSS patients.

Characteristics	pSS (*n* = 133)
Demographic characteristics	
Age (years, mean ± SD)	48.3 ± 15.0
Sex (female), *n* (%)	127/133 (96%)
Disease duration (years), median (IQR)	4 (1–8)
Key clinical manifestations	
Xerostomia, *n* (%)	103/133 (77%)
Xerophthalmia, *n* (%)	79/133 (59%)
Fatigue, *n* (%)	27/133 (20%)
System involvement	
Hematological, *n* (%)	30/133 (23%)
Cutaneous, *n* (%)	26/133 (20%)
Articular, *n* (%)	19/133 (14%)
Liver, *n* (%)	17/133 (13%)
Pulmonary, *n* (%)	14/133 (11%)
Renal, *n* (%)	12/133 (9%)
Peripheral nervous system, *n* (%)	3/133 (2.3%)
Laboratory examinations	
ANA positive (≥1:80), *n* (%)	131/133 (98.4%)
Anti‐SSA positive, *n* (%)	114/133 (86%)
Anti‐Ro52 positive, *n* (%)	87/108 (81%)
Anti‐SSB positive, *n* (%)	56/133 (42%)
IgG elevated (>16 g/L), *n* (%)	67/130 (52%)
RF (IU/mL), median (IQR)	54 (18–122)
ESR (mm/h), median (IQR)	20 (11–29)
Scoring	
ESSPRI	2 (0–5)
ESSDAI	2 (0–5)
SSDDI	2 (2–3)

Abbreviations: ANA, anti‐nuclear antibodies; ESR, erythrocyte sedimentation rate; ESSDAI, EULAR Sjögren’s syndrome disease activity index; ESSPRI, EULAR Sjögren’s syndrome patient reported index; IQR, interquartile range; MSGB, minor salivary gland biopsy; RF, rheumatoid factor; SD, standard deviation; SSDDI, Sjögren’s syndrome disease damage index.

Laboratory tests detected anti‐SSA antibodies in 86% of patients, with anti‐Ro52 and anti‐SSB antibodies present in 81% and 42% of cases, respectively. The most prevalent immunoglobulin abnormality was elevated IgG levels, occurring in 52% of patients. The median rheumatoid factor (RF) and erythrocyte sedimentation rate (ESR) were 54 (18–122) IU/mL and 20 (11–29) mm/h, respectively.

### 3.2. Distinct Lymphocyte Profile in Patients With pSS and Healthy Volunteers

To assess the alterations in patterns of peripheral blood lymphocytes in pSS, both the number and percentage of lymphocyte subsets were examined in 133 pSS patients and 241 sex‐ and age‐matched HCs (230 females and 11 males, age 48.5 ± 14.3 years). Analysis using the Mann–Whitney *U* test and univariate logistic regression showed that the proportions of CD8^+^ T cells, CD38^+^CD8^+^ T cells, and HLA‐DR^+^CD8^+^ T cells in pSS patients were significantly higher than those in HCs. In contrast, the proportions of CD4^+^ T cells were decreased, resulting in an imbalance in the CD4/CD8 ratio. The proportions of CD28^+^CD4^+^ T cells and CD28^+^CD8^+^ T cells were significantly reduced in pSS. A notable decrease was observed in the frequency of naïve CD4^+^ T cells in pSS patients, while the frequency of memory CD4^+^ T cells was elevated significantly. Furthermore, the percentage of NK cells was significantly decreased. No significant difference was found in the proportion of B cells (Figure 1A, B). For further validation, we performed a sPLS‐DA model. The results indicated a partial overlap between the lymphocyte profiles of patients with pSS and HCs (Figure 1C). The percentage of CD38^+^CD8^+^ T cells, HLA‐DR^+^CD8^+^ T cells, and memory CD4^+^ T cells were among the top contributors to the segregation of patients with pSS from HCs in the sPLS‐DA model (Figure 1D).

Figure 1Comparative analysis of lymphocyte profiles between HCs (*n* = 241) and pSS (*n* = 133). (A) Violin‐box plots: Distribution of lymphocyte subset frequencies in HCs and pSS. Data were analyzed using Mann–Whitney *U*‐tests. (B) Forest plot displaying univariate logistic regression analysis of 12 immunological parameters, showing odds ratios (ORs) with 95% confidence intervals. (C) sPLS‐DA plot with confidence ellipses (green = HCs and orange = pSS). (D) Component 1 factor loading weights for top 10 immunological parameters (green = HCs and orange = pSS). CD28T4, CD28^+^CD4^+^ T cells; CD28T8, CD28^+^CD8^+^ T cells; CD38T8, CD38^+^CD8^+^ T cells; DRT8, HLA‐DR^+^CD8^+^ T cells; HC, healthy controls; MeT4, memory CD4^+^ T cells; NaT4, naive CD4^+^ T cells; NK, natural killer cells; sPLS‐DA, sparse partial least squares discriminant analysis; pSS, primary Sjögren’s syndrome; T4, CD4^+^ T cells; T8, CD8^+^ T cells.  ^∗^ *p* < 0.05,  ^∗∗^ *p* < 0.01,  ^∗∗∗^ *p* < 0.001.

**Figure:**
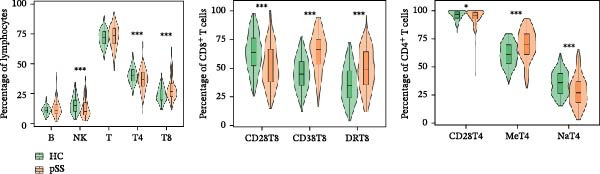
(A)

**Figure:**
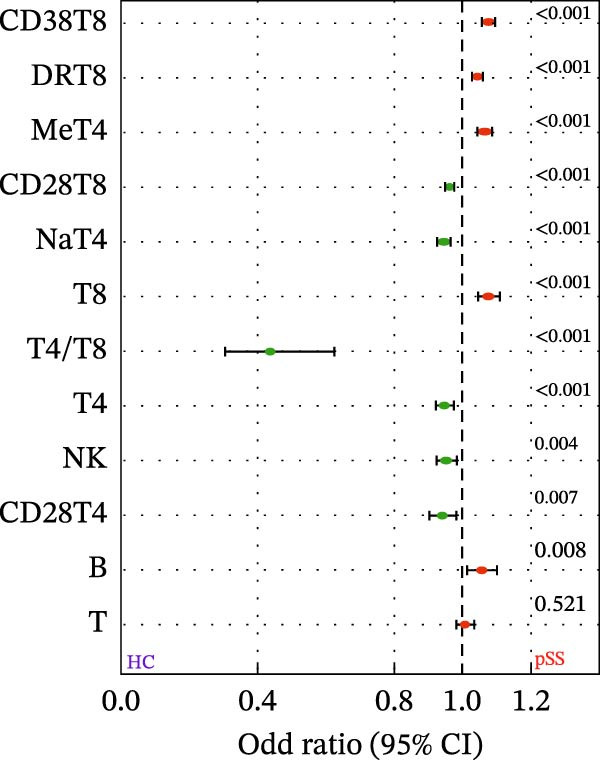
(B)

**Figure:**
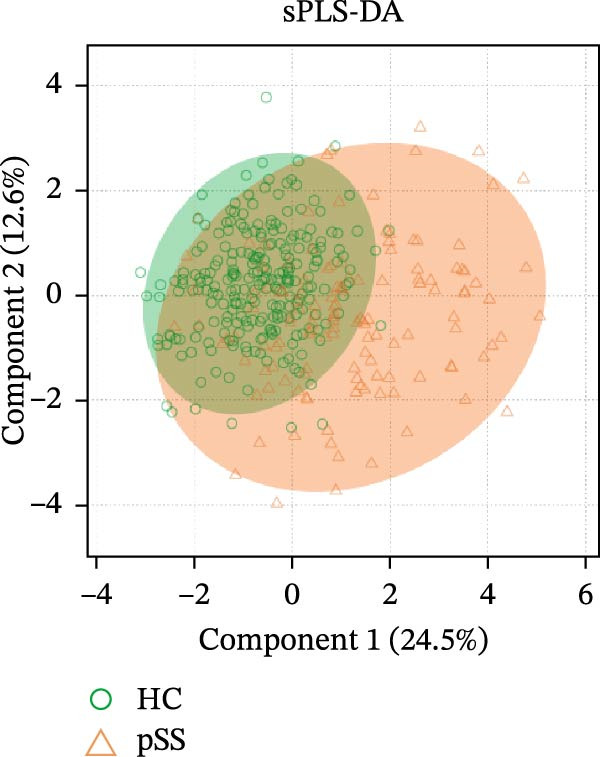
(C)

**Figure:**
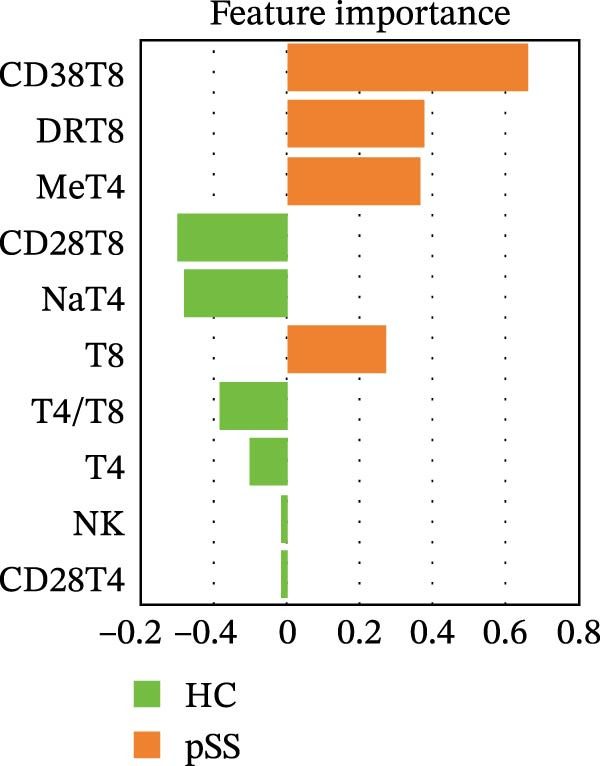
(D)

Consistent with the alterations in lymphocyte subset proportions, the estimated counts of several subsets also differed between pSS patients and HCs. Specifically, we observed higher counts of CD8^+^ T cells, CD38^+^CD8^+^ T cells, and HLA‐DR^+^CD8^+^ T cells, and lower counts of CD4^+^ T cells, naïve CD4^+^ T cells, CD28^+^CD4^+^ T cells, and NK cells (Supporting Information 1: Figure S3). Taken together, these comparative analyses indicate that patients with pSS exhibit a distinct immune phenotype compared to HCs.

### 3.3. Stratification of pSS Patients Into Three Distinct Subgroups Based on Lymphocyte Profile

To address heterogeneity in lymphocyte subset alterations among pSS patients, we performed *K*‐means clustering after confirming the optimal number of clusters as three (Supporting Information 1: Figure S4A). This analysis identified three distinct lymphocyte‐driven subgroups (Figure 2A). Cluster 1 (*n* = 30) was distinguished by significantly higher proportions of CD8^+^ T cells, CD38^+^CD8^+^ T cells, and a pronounced CD4/CD8 imbalance (Figure 2B). Cluster 2 (*n* = 33) featured higher proportions of NK cells and B cells than other clusters. Notably, Cluster 1 and Cluster 2 shared similar lymphocyte signatures, featuring reduced proportions of CD4^+^ T cells, CD28^+^CD4^+^ T cells, and CD28^+^CD8^+^ T cells alongside elevated proportions of memory CD4^+^ T cells and HLA‐DR^+^CD8^+^ T cells. As the sPLS‐DA plot shows (Figure 2C), Cluster 3, the largest group with 70 patients, had lymphocyte distributions that most closely resembled those of HCs, albeit with a reduced percentage of NK cells and an increased percentage of CD38^+^CD8^+^ T cells (Figure 2B). Additionally, total lymphocyte counts did not differ significantly between clusters. Both the estimated counts and frequencies of lymphocyte subsets displayed similar distribution patterns among the three clusters (Supporting Information 1: Figure S4B).

Figure 2Cluster analysis based on the lymphocyte subsets in pSS. (A) *K*‐means clustering (*k* = 3) visualization. (B) Lymphocyte subset distribution across Cluster 1 (C1, *n* = 30), Cluster 2 (C2, *n* = 33), Cluster 3 (C3, *n* = 70), and HC group (*n* = 241), presented as median with interquartile range. (C) sPLS‐DA plot showing the three clusters identified among pSS patients and HC group. CD28T4, CD28^+^CD4^+^ T cells; CD28T8, CD28^+^CD8^+^ T cells; CD38T8, CD38^+^CD8^+^ T cells; DRT8, HLA‐DR^+^CD8^+^ T cells; HC, healthy controls; MeT4, memory CD4^+^ T cells; NaT4, naive CD4^+^ T cells; NK, natural killer cells; sPLS‐DA, sparse partial least squares discriminant analysis; pSS, primary Sjögren’s syndrome; T4, CD4^+^ T cells; T8, CD8^+^ T cells.  ^∗^Comparisons among three pSS clusters. ^#^Comparisons between pSS clusters and HC group.  ^∗^ *p* < 0.05,  ^∗∗^ *p* < 0.01,  ^∗∗∗^ *p* < 0.001;  ^#^ *p* < 0.05,  ^##^ *p* < 0.01,  ^###^ *p* < 0.001. Data were analyzed using the Kruskal–Wallis test, followed by Dunn’s multiple comparisons test.

**Figure:**
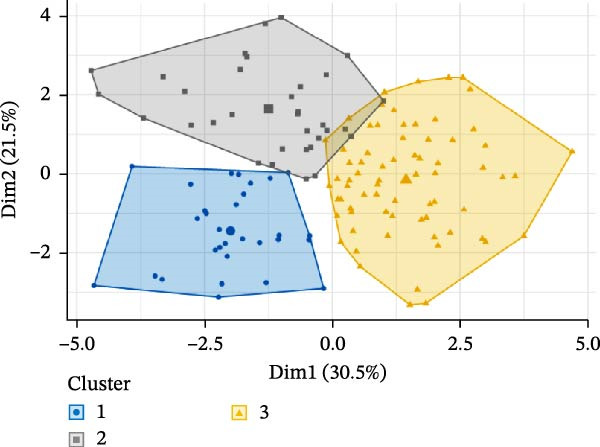
(A)

**Figure:**
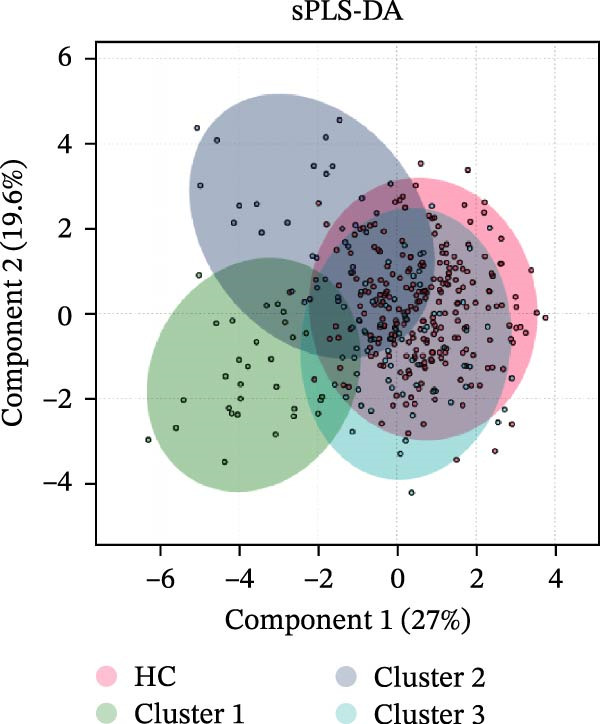
(B)

**Figure:**
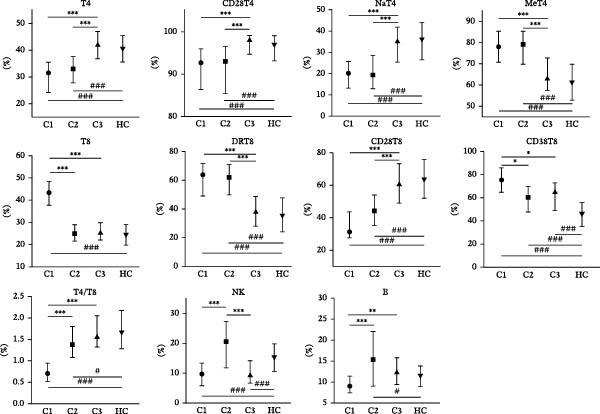
(C)

### 3.4. Distinct Clinical Characteristics Across Three Clusters

To assess whether the distinct immunologic profiles correlated with clinical heterogeneity, we compared disease manifestations, laboratory markers, disease activity, and damage scores across the three clusters (Table 2). Cluster 1 showed a trend toward a higher prevalence of hematologic abnormalities, including anemia and thrombocytopenia (though statistically nonsignificant), alongside comparable other systemic involvement relative to Clusters 2 and 3 (Figure 3A). Notably, this cluster displayed marked serologic activity, with significantly higher rates of hyperglobulinemia, anti‐Ro52 positivity, and high‐titer ANA positivity (Figure 3B).

Figure 3Clinical characteristics among the identified pSS clusters: Cluster 1 (C1, *n* = 30), Cluster 2 (C2, *n* = 33), and Cluster 3 (C3, *n* = 70). (A) Clinical profile of three clusters. (B) Serological profile of three clusters. (C) SSDDI grading of three clusters. ANA, anti‐nuclear antibody; ESR, erythrocyte sedimentation rate; RF, rheumatoid factor; SSDDI, Sjögren’s syndrome disease damage index.  ^∗^ *p* < 0.05,  ^∗∗^ *p* < 0.01. Data were analyzed using chi‐square test or Fisher’s exact test.

**Figure:**
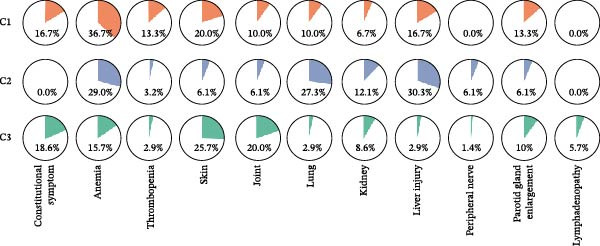
(A)

**Figure:**
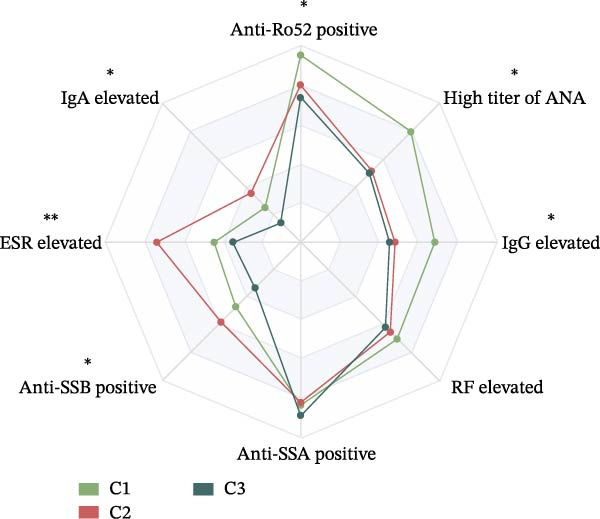
(B)

**Figure:**
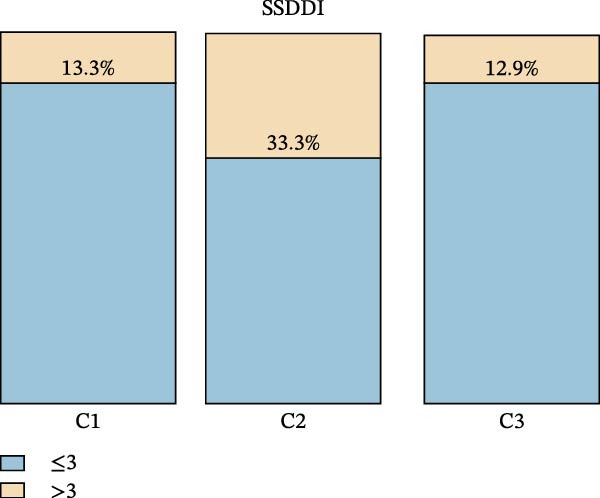
(C)

**Table 2:** Clinical characteristics between clusters.

Characteristics	C1 (*n* = 30)	C2 (*n* = 33)	C3 (*n* = 70)	*p*‐Value
Demographic characteristics				
Age (years), mean ± SD	46.0 ± 13.4	57.0 ± 13.6	45.2 ± 14.9	<0.001
Sex, female, *n* (%)	30/30 (100%)	29/33 (88%)	68/70 (97%)	0.06
Disease duration (years), median (IQR)	5 (3–9)	4 (1–8)	3 (1–9)	0.38
Clinical manifestations				
Xerostomia, *n* (%)	23/30 (77%)	28/33 (85%)	52/70 (74%)	0.52
Xerophthalmia, *n* (%)	16/30 (53%)	24/33 (73%)	39/70 (56%)	0.20
Fatigue, *n* (%)	7/30 (23%)	3/33 (9%)	17/70 (24%)	0.18
Constitutional symptom, *n* (%)	5/30 (17%)	0/33 (0%)	13/70 (19%)	0.01
Parotid gland enlargement, *n* (%)	4/30 (13%)	2/33 (6%)	7/70 (10%)	0.63
Raynaud, *n* (%)	1/30 (3%)	1/33 (3%)	1/70 (1.4%)	0.60
Lymphadenopathy, *n* (%)	0/30 (0%)	0/33 (0%)	4/70 (6%)	0.18
System involvement				
Cutaneous, *n* (%)	6/30 (20%)	2/33 (6%)	18/70 (26%)	0.06
Articular, *n* (%)	3/30 (10%)	2/33 (6%)	14/70 (20%)	0.15
Pulmonary, *n* (%)	3/30 (10%)	9/33 (27%)	2/70 (3%)	<0.001
Renal, *n* (%)	2/30 (7%)	4/33 (12%)	6/70 (9%)	0.78
Liver, *n* (%)	5/30 (17%)	10/33 (30%)	2/70 (3%)	<0.001
Peripheral nervous system, *n* (%)	0/30 (0%)	2/33 (6%)	1/70 (1%)	0.28
Laboratory examinations				
ANA ≥1:320, *n* (%)	24/30 (80%)	17/33 (52%)	35/70 (54%)	0.02
Anti‐SSA positive, *n* (%)	25/30 (83%)	27/33 (82%)	62/70 (89%)	0.54
Anti‐Ro52 positive, *n* (%)	22/23 (96%)	25/31 (81%)	40/54 (74%)	0.09
Anti‐SSB positive, *n* (%)	14/30 (47%)	19/33 (58%)	23/70 (33%)	0.05
IgG (g/L), mean ± SD	19.9 ± 7.5	17.1 ± 5.6	16.1 ± 4.8	0.01
IgA (g/L), mean ± SD	3.4 ± 1.7	3.6 ± 1.6	2.9 ± 1.1	0.03
IgM (g/L), mean ± SD	1.5 ± 1.0	1.3 ± 0.7	1.2 ± 0.7	0.37
RF (IU/mL), median (IQR)	43 (19–368)	44 (16–111)	67 (20–112)	0.71
C3 (g/L), mean ± SD	0.98 ± 0.17	1.08 ± 0.24	1.02 ± 0.18	0.18
C4 (g/L), mean ± SD	0.17 ± 0.08	0.19 ± 0.07	0.18 ± 0.05	0.45
hs‐CRP (mg/L), median (IQR)	0.75 (0.47–2.10)	1.38 (0.43–2.55)	0.96 (0.34–1.69)	0.38
ESR, (mm/h), median (IQR)	19 (12–32)	26 (19–35)	14 (9–24)	<0.001
Lymphopenia (<1 × 10^9^/L), *n* (%)	6/30 (20%)	7/33 (21%)	18/70 (26%)	0.77
Neutropenia (<1.5 × 10^9^/L), *n* (%)	6/30 (20%)	10/33 (30%)	26/70 (37%)	0.22
Anemia (<120 g/L), *n* (%)	11/30 (37%)	9/31 (29%)	11/70 (16%)	0.06
Thrombopenia (<100 × 10^9^/L), *n* (%)	4/30 (13%)	1/31 (3%)	2/70 (3%)	0.10
Scoring, median (IQR)				
ESSPRI	2 (0–6)	2 (0–4)	2 (0–5)	0.82
ESSDAI	2 (1–5)	2 (1–5)	2 (0–5)	0.49
SSDDI	2 (2–3)	3 (2–4)	2 (1–3)	0.002

*Note:* Statistical tests performed: chi‐square test of independence for categorical variables and Kruskal–Wallis test for continuous variables.

Abbreviations: C3, complement 3; C4, complement 4; hs‐CRP, high‐sensitivity C‐reactive protein.

Cluster 2 was distinguished by higher SSDDI damage scores (Figure 3C). This group presented with predominant involvement of parenchymal organs, including the lung and liver, along with trends toward higher renal and peripheral nerve involvement, features that aligned with their higher damage scores. In contrast, constitutional symptoms, cutaneous manifestations, and joint involvement were reported least frequently in Cluster 2. Laboratory findings included elevated IgA, increased ESR, and more frequent anti‐SSB positivity. Additionally, Cluster 2 had the oldest average age among all clusters. Cluster 3 showed the highest prevalence of constitutional symptoms, articular and cutaneous manifestations, while exhibiting the lowest rates of pulmonary and hepatic involvement. Serologically, this group displayed the lowest positivity rates for anti‐SSB and anti‐Ro52 antibodies, along with significantly reduced ESR, IgG, and IgA levels. Despite these phenotypic differences, disease activity scores (ESSDAI and ESSPRI) were comparable across the three clusters (Table 2).

## 4. Discussion

Our study offers a comprehensive analysis of immunological profiles of pSS patients, characterizing distinct alterations in lymphocyte subsets. We identified that activated CD8^+^ T cell subsets, including CD38^+^CD8^+^ T cells and HLA‐DR^+^CD8^+^ T cells were among the key features distinguishing patients from healthy individuals. Cluster 1, characterized predominantly by elevated CD8^+^ T cells, showed prominent serological abnormalities and balanced systemic involvement. In line with this, serum immunoglobulin levels have been reported to correlate with the proportion of HLA‐DR^+^CD8^+^ T cells in pSS [32]. Mingueneau et al. [21] reported abundant activated HLA‐DR^+^CD8^+^ T cells in labial glands correlating with disease activity but showed no significant association with CD4^+^ T cells. Mechanistically, CD8^+^ T cells are actively recruited into salivary gland tissues, where they may promote epithelial apoptosis via cytotoxic T lymphocyte (CTL)‐mediated mechanisms or the Fas‐FasL pathway [32]. Importantly, murine studies revealed CD8^+^ T cell depletion not CD4^+^ T cell restored salivary function, underscoring their potential central role in glandular destruction [33]. We also observed a significant naive‐memory T cell imbalance and elevated CD28 null T cell populations in pSS patients, suggestive of accelerated immunosenescence in pSS [34, 35]. Senescent T cells are recognized as a risk determinant in rheumatoid arthritis and giant cell arteritis [36, 37]. Notably, this premature immunosenescence, coupled with CD4^+^ T cell deficiency, could thus contribute to the increased infection susceptibility frequently observed in pSS patients [38, 39].

Our analysis indicated a reduction in both the estimated count and proportion of NK cells in the peripheral blood of pSS patients. However, previous studies have reported inconsistent findings regarding NK cell alterations in pSS [40–42]. These discrepancies could be attributable to the high heterogeneity of enrolled patient cohorts. In our cohort, we identified a subgroup without significant NK cell reduction, which was associated with a higher disease damage index. Such heterogeneity in NK cells among pSS patients has also been observed in other studies. Low peripheral NK cell numbers have been associated with a better response to B‐cell‐targeted therapy in pSS [43], whereas patients with elevated NK cell levels often exhibit a strong type II IFN signature—rather than a B‐cell activation–related type I IFN signature [44].

This study employed unsupervised machine learning to stratify pSS patients into three distinct subgroups based on lymphocyte profiling, which helped delineate associations between immune signatures and clinical manifestations. Cluster 1 was characterized by pronounced CD8^+^ T cell expansion, hematologic involvement, and heightened serological activity (e.g., anti‐Ro52 positivity and hyperglobulinemia). Previous studies have shown that IFN pathway activation enhances and sustains cytotoxic function in CD8^+^ T lymphocytes [45, 46]. Transcriptomic analyses have similarly identified an IFN‐driven subset in pSS, where type I IFN signatures correlate with hematologic and serological abnormalities [11]. This aligns with the profile of Cluster 1, suggesting it may represent a subtype enriched for IFN‐related signaling. Additionally, the markedly reduced CD4/CD8 ratio in Cluster 1 is reminiscent of the immune signature of group 2 from the Martin‐Gutierrez et al. [47] pSS classification. Although Cluster 1 and Cluster 2 exhibited similar lymphocyte abnormalities—primarily CD4^+^ T cell dysregulation, reflected by reduced CD28^+^ T cells, and altered naive/memory CD4^+^ T cell ratios—they presented distinct clinical profiles despite sharing high serological activity. These observations suggest that while CD4^+^ T cell dysregulation is a recurrent immunological feature in pSS, it might not be the sole driver of disease progression in all patient subsets. This divergence could help explain why some targeted T cell therapies, such as abatacept, have shown limited clinical efficacy in pSS despite inducing measurable biological effects [5].

Cluster 2 exhibited higher NK and B cells than the other clusters, along with increased organ damage (pulmonary and hepatic) and higher SSDDI scores. Even in mild‐to‐moderate cases, Cluster 2 showed evidence of significant organ damage accumulation, which may warrant closer monitoring, potentially including HRCT, renal function assessment, and liver elastography to help prevent irreversible progression. Notably, the elevated IgA levels in this subgroup were consistent with reports linking IgA to pulmonary involvement [48]. IgA‐containing immune complexes (CIC) are thought to play a role in mediating the tissue injury associated with pSS [49]. Cluster 3, the largest identified subgroup, exhibited lymphocyte profiles and minimal serological activity most closely resembled those of HCs, which aligns it with the “healthy‐like” populations described in other pSS classification studies. These populations are typically characterized by low ESSDAI scores and an absence of anti‐SSA antibodies [11], or by the lack of IFN signature and inflammatory gene activation [50]. Clinically, Cluster 3 was marked by skin and joint involvement but showed the least parenchymal organ damage. However, the seemingly benign peripheral blood profile in such subgroups may not accurately reflect the state of tissue infiltration. Therefore, future studies could assess glandular infiltration in these subgroups to better understand their pathological features.

Several limitations must be acknowledged. First, the predominance of patients with mild‐to‐moderate disease activity may limit the generalizability of our findings, and we were unable to evaluate the impact of high disease activity on the identified immune signatures. Second, larger validation cohorts are needed to confirm the robustness of our clustering approach and to determine whether these immune phenotypes reflect stable endotypes or transient disease states. Third, while our data show distinct clinical characteristics across the clusters, their longitudinal outcomes (e.g., lymphoma‐free survival) remain unaddressed. Future work is needed to evaluate the prognostic implications during follow‐up. Finally, although our analysis provided insights into immune dysregulation, more comprehensive immune profiling combined with multiomics analyses could reveal deeper biological differences between clusters.

In summary, this study suggests three immunophenotypic clusters in pSS, each linked to a distinct clinical profile. These findings underscore the heterogeneity of pSS and may provide a basis for future research on patient stratification.

## Author Contributions

Yimeng Jia and Sicheng Huang collected and analyzed the data and wrote the original draft. Ye Guo, Anqi Wang, and Chuiwen Deng designed the study, rechecked the diagnosis of all patients, and revised the manuscript. Yunyun Fei contributed to the conceptualization, funding acquisition, and finalization of the manuscript.

## Funding

This work was supported by the Beijing Natural Science Foundation Program (Grant 7242103), the National Natural Science Foundation of China (Grant 82572065), the National High Level Hospital Clinical Research Funding (Grant 2025‐PUMCH‐C‐039 and 2022‐PUMCH‐C‐039), and the CAMS Innovation Fund for Medical Sciences, CIFMS (Grant 2023‐I2M‐C&T‐B‐006).

## Disclosure

This manuscript was approved by all authors for publication.

## Ethics Statement

The study was approved by the institutional review board of PUMCH (I‐24PJ1657). The ethics committee waived the requirement of written informed consent for participation.

## Conflicts of Interest

The authors declare no conflicts of interest.

## Supporting Information

Additional supporting information can be found online in the Supporting Information section.

## Supporting information


**Supporting Information 1** Figure S1. Flow cytometric gating strategy for the analysis of lymphocyte subsets. Figure S2. Balanced error rate (BER) from 10 repeats of five‐fold cross‐validation for evaluating the performance of the sPLS‐DA model. Figure S3. Differential analysis of estimated lymphocyte subset counts between primary Sjögren’s syndrome (pSS) and healthy controls (HCs). (A) Volcano plot displaying significant differences in the estimated number of lymphocyte subsets between pSS and HCs. (B) Violin plot displaying the estimated number of lymphocyte subsets that were significantly different between pSS and HCs. CD38T8: CD38^+^CD8^+^ T cells; DRT8: HLA‐DR^+^CD8^+^ T cells; CD28T8: CD28^+^CD8^+^ T cells; CD28T4: CD28^+^CD4^+^ T cells; NaT4: naive CD4^+^ T cells; and MeT4: memory CD4^+^ T cells.  ^∗^ *p* < 0.05,  ^∗∗^ *p* < 0.01,  ^∗∗∗^ *p* < 0.001, and  ^∗∗∗∗^ *p* < 0.0001. Data were analyzed using Mann–Whitney *U*‐tests. Figure S4. (A) Determination of optimal cluster number. The elbow plot shows the within‐cluster sum of squares (WSS) versus cluster number (*k*). The chosen *k* (red dashed line) corresponds to the “elbow” point. (B) Lymphocyte subset counts across Cluster 1 (C1, *n* = 30), Cluster 2 (C2, *n* = 33), and Cluster 3 (C3, *n* = 70) are presented as median (interquartile range).  ^∗^ *p* < 0.05,  ^∗∗^ *p* < 0.01,  ^∗∗∗^ *p* < 0.001,  ^∗∗∗∗^ *p* < 0.0001. Data were analyzed using the Kruskal–Wallis test, followed by Dunn’s multiple comparisons test. Table S1. Supplementary clinical characteristics of pSS patients. Method details: Method details of sPLS‐DA validation and *k*‐means clustering in R.

**Supporting Information 2** Data table: Lymphocyte subsets in pSS patients and HC.

## Data Availability

The data generated during this study are available within the article or its supporting information section.
