# Supplementary material for: Immunophenotypic Stratification of Primary Sjögren’s Syndrome Reveals Distinct Lymphocyte Profiles and Clinical Manifestations
Source: J Immunol Res. 2026 Feb 26;2026:9295560. doi: 10.1155/jimr/9295560 (PMC13140272; doi:10.1155/jimr/9295560)
Supplement: Supplementary file 1 — Supporting Information 1 Figure S1. Flow cytometric gating strategy for the analysis of lymphocyte subsets. Figure S2. Balanced error rate (BER) from 10 repeats of five‐fold cross‐validation for evaluating the performance of the sPLS‐DA model. Figure S3. Differential analysis of estimated lymphocyte subset counts between primary Sjögren’s syndrome (pSS) and healthy controls (HCs). (A) Volcano plot displaying significant differences in the estimated number of lymphocyte subsets between pSS and HCs. (B) Violin plot displaying the estimated number of lymphocyte subsets that were significantly different between pSS and HCs. CD38T8: CD38+CD8+ T cells; DRT8: HLA‐DR+CD8+ T cells; CD28T8: CD28+CD8+ T cells; CD28T4: CD28+CD4+ T cells; NaT4: naive CD4+ T cells; and MeT4: memory CD4+ T cells. ∗ p < 0.05, ∗∗ p < 0.01, ∗∗∗ p < 0.001, and ∗∗∗∗ p < 0.0001. Data were analyzed using Mann–Whitney U‐tests. Figure S4. (A) Determination of optimal cluster number. The elbow plot shows the within‐cluster sum of squares (WSS) versus cluster number (k). The chosen k (red dashed line) corresponds to the “elbow” point. (B) Lymphocyte subset counts across Cluster 1 (C1, n = 30), Cluster 2 (C2, n = 33), and Cluster 3 (C3, n = 70) are presented as median (interquartile range). ∗ p < 0.05, ∗∗ p < 0.01, ∗∗∗ p < 0.001, ∗∗∗∗ p < 0.0001. Data were analyzed using the Kruskal–Wallis test, followed by Dunn’s multiple comparisons test. Table S1. Supplementary clinical characteristics of pSS patients. Method details: Method details of sPLS‐DA validation and k‐means clustering in R. [file JIMR-2026-9295560-s001.docx]

**Table S1. Supplementary clinical characteristics of pSS patients.**

| Characteristics |  |
| --- | --- |
| **Clinical manifestations** |  |
| Constitutional symptom, n (%) | 18/133 (13.5%) |
| Parotid gland enlargement, n (%) | 13/133 (9.8%) |
| Lymphadenopathy, n (%) | 4/133 (3.0%) |
| Raynaud, n (%) | 3/133 (2.3%) |
| **Laboratory examinations** |  |
| MSGB, Lymphocytic focus≥1, n (%) | 30/30 (100.0%) |
| uSFR (mL/ min), median (IQR) | 0.06 (0.01–0.08) |
| BUT (s), median (IQR) | 2 (1-6) |
| Schirmer test (mm/5 min), median (IQR) | 6 (2-14) |
| IgA, g/L, mean±SD | 3.2**±**1.4 |
| IgM, g/L, mean±SD | 1.3**±**0.8 |
| Lymphopenia (<1×10^9^/L), n (%) | 31/133 (23.3%) |
| Neutropenia (<1.5×10^9^/L), n (%) | 42/133 (31.6%) |
| Anemia (<120g/L), n (%) | 31/131 (23.7%) |
| Thrombopenia (<100×10^9^/L), n (%) | 7/131 (5.3%) |

MSGB, Minor Salivary Gland Biopsy; BUT, Tear Film Break-up Time.

**Figure S1:** **Flow cytometric gating strategy for the analysis of lymphocyte subsets.**

**
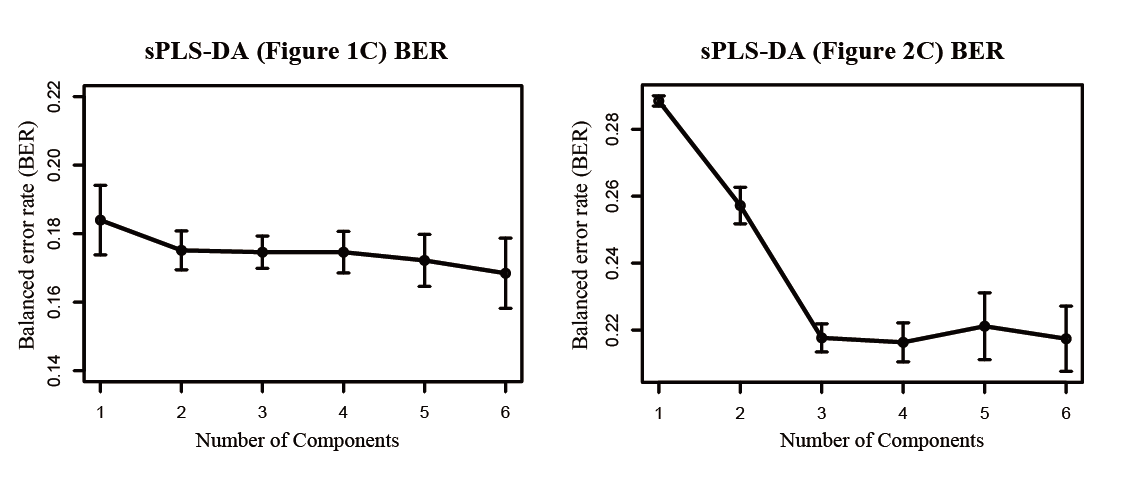
**

**Figure S2: Balanced error rate (BER) from 10 repeats of 5-fold cross-validation for evaluating the performance of the sPLS-DA model.**
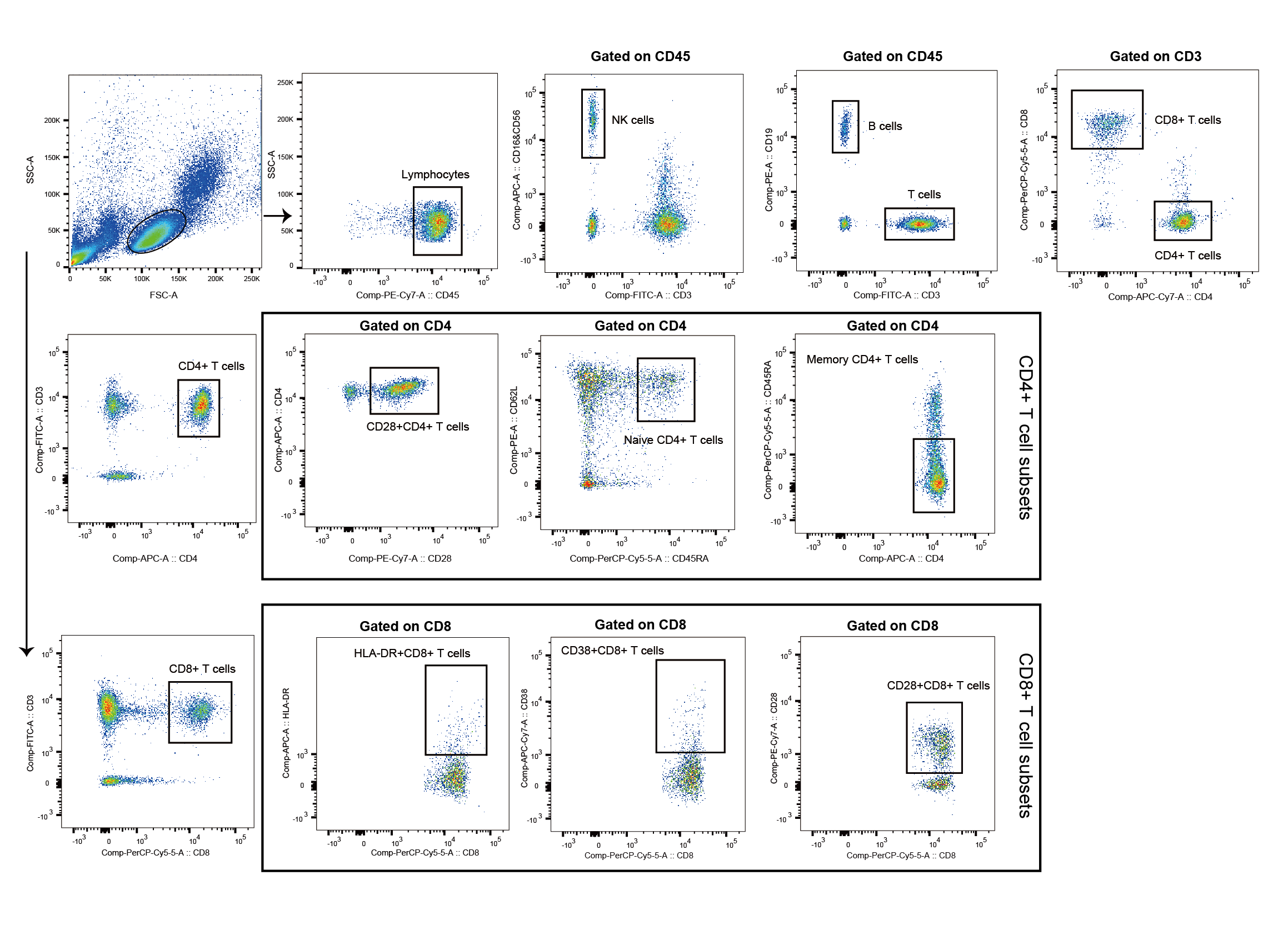


**
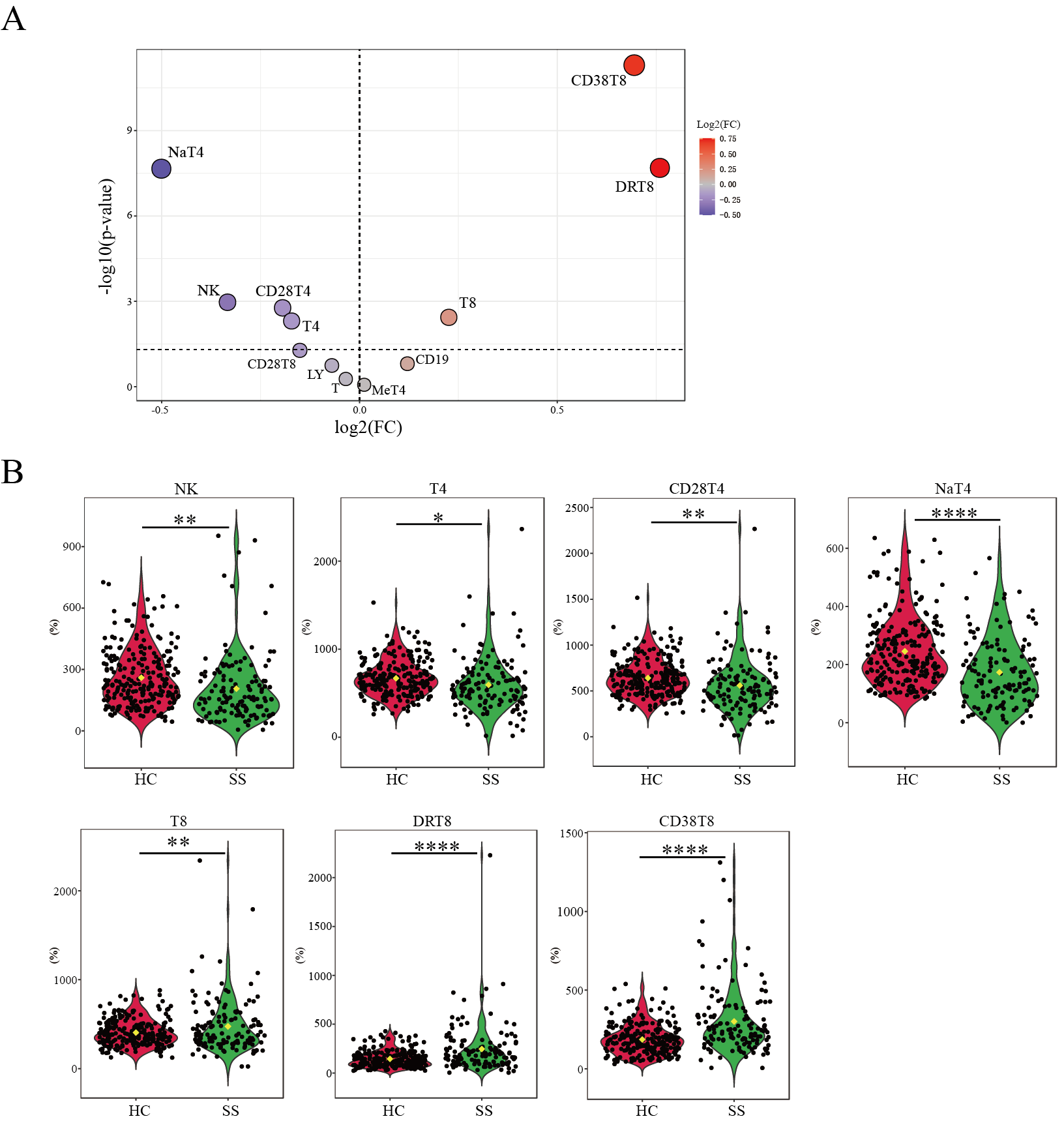
**

**Figure S3:** **Differential analysis of estimated lymphocyte subset counts between primary**

**Sjögren's syndrome (pSS) and healthy controls (HCs).**

1. Volcano plot displaying significant differences in the estimated number of lymphocyte subsets between pSS and HCs. (B) Violin plot displaying the estimated number of lymphocyte subsets that were significantly different between pSS and HCs. CD38T8: CD38^+^CD8^+^T cells; DRT8: HLA-DR^+^CD8^+^T cells; CD28T8: CD28^+^CD8^+^T cells; CD28T4: CD28^+^CD4^+^T cells; NaT4: naive CD4^+^T cells; MeT4: memory CD4^+^T cells. *=p<0.05, **=p<0.01, ***=p<0.001, ****=p<0.0001. Data were analyzed using Mann–Whitney U-tests.


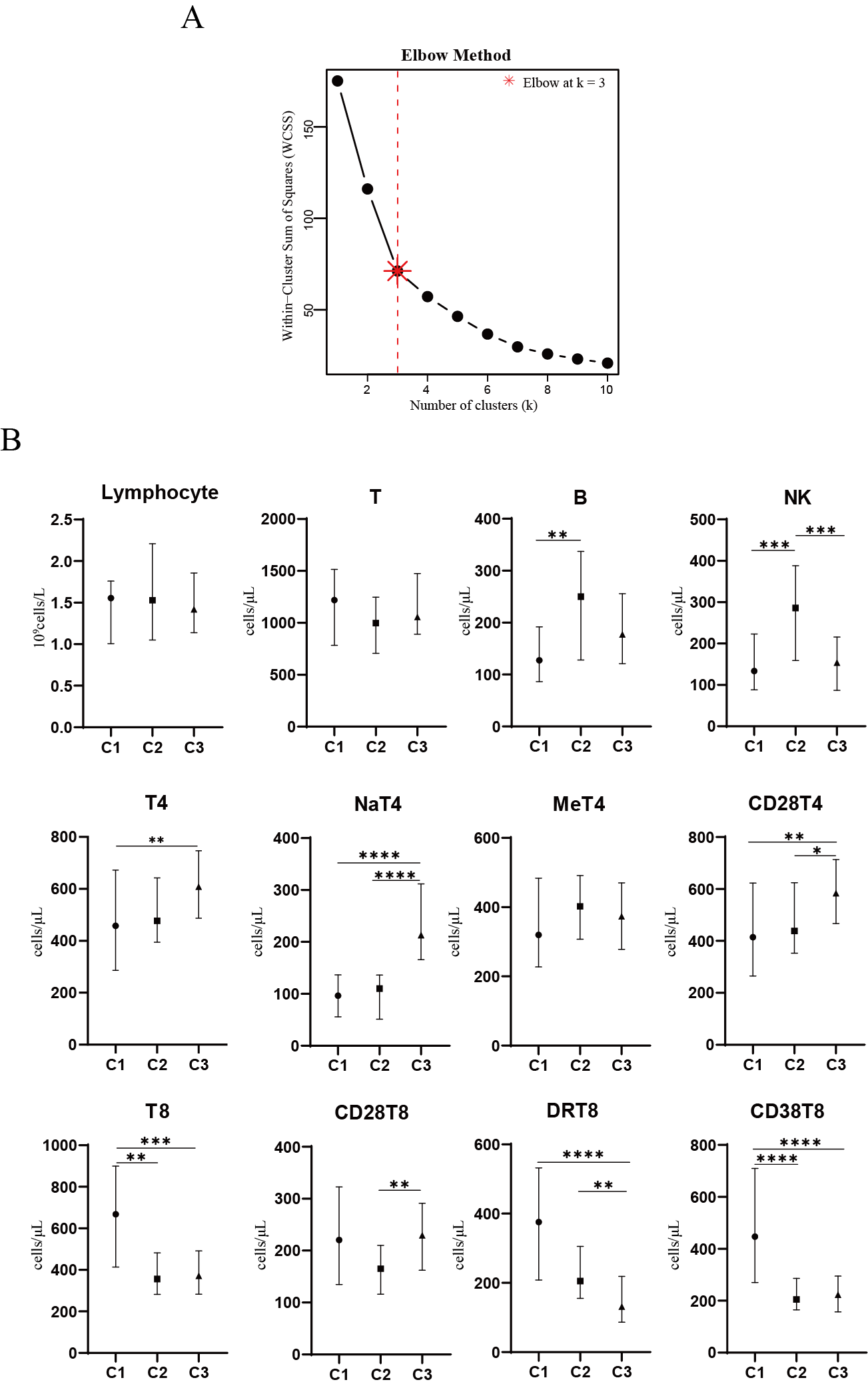


**Figure S4:** (A) Determination of optimal cluster number. The elbow plot shows the within-cluster sum of squares (WSS) versus cluster number (k). The chosen k (red dashed line) corresponds to the "elbow" point. (B) Lymphocyte subset counts across Cluster 1 (C1, n=30), Cluster 2 (C2, n=33), and Cluster 3 (C3, n=70) are presented as median (interquartile range). *p<0.05, **p<0.01, ***p<0.001, ****p<0.0001. Data were analyzed using the Kruskal-Wallis test, followed by Dunn's multiple comparisons test.

**Method details**

**sPLS-DA validation**

#library packages needed

library(mixOmics)

library(BiocParallel)

# Parameter tuning

set.seed(123)

bpparam <- SnowParam(workers = 2)

tune.splsda.result <- tune.splsda(

X = X, Y = Y, ncomp = min(6, ncol(X)),

validation = 'Mfold', folds = 5, nrepeat = 10,

dist = 'max.dist', measure = "BER",

test.keepX = list.keepX, BPPARAM = bpparam)

# Optimal parameters

ncomp <- tune.splsda.result$choice.ncomp$ncomp

select.keepX <- tune.splsda.result$choice.keepX[1:ncomp]

# Final model

final.splsda <- splsda(X = X, Y = Y, ncomp = ncomp, keepX = select.keepX)

# Performance validation

perf.splsda <- perf(final.splsda, validation = "Mfold",

folds = 5, nrepeat = 10, dist = "max.dist")

**k-means clustering in R**

#library packages needed

library(factoextra)

library(ggplot2)

library(cluster)

library(NbClust)

#data normalization

mat.scaled<-scale(mat)

#determine the optimal number of clustering

k_elbow <- fviz_nbclust(mat.scaled, kmeans, method = "wss")

#k-means clustering

set.seed(123)

km <- kmeans(mat.scaled, centers = 3, nstart = 50)

p <- fviz_cluster(km, data = mat.scaled,

palette = "jco",

ggtheme = theme_minimal())
